# Detecting intermediate protein conformations using algebraic topology

**DOI:** 10.1186/s12859-017-1918-z

**Published:** 2017-12-06

**Authors:** Nurit Haspel, Dong Luo, Eduardo González

**Affiliations:** 1 Department of Computer Science, University of Massachusetts Boston, 100 Morrissey Blvd., Boston, 02125 MA USA; 2 Department of Mathematics, University of Massachusetts Boston, 100 Morrissey Blvd., Boston, 02125 MA USA

**Keywords:** Algebraic topology, Protein conformational sampling, Clustering, Protein structure, Dimensionality reduction

## Abstract

**Background:**

Understanding protein structure and dynamics is essential for understanding their function. This is a challenging task due to the high complexity of the conformational landscapes of proteins and their rugged energy levels. In particular, it is important to detect highly populated regions which could correspond to intermediate structures or local minima.

**Results:**

We present a hierarchical clustering and algebraic topology based method that detects regions of interest in protein conformational space. The method is based on several techniques. We use coarse grained protein conformational search, efficient robust dimensionality reduction and topological analysis via persistent homology as the main tools. We use two dimensionality reduction methods as well, robust Principal Component Analysis (PCA) and Isomap, to generate a reduced representation of the data while preserving most of the variance in the data.

**Conclusions:**

Our hierarchical clustering method was able to produce compact, well separated clusters for all the tested examples.

## Background

Characterizing the conformational space of proteins is crucial for understanding the way they perform their function. Understanding the connection between protein structure, dynamics and function can contribute substantially to our understanding of cellular processes involving proteins. The question of how the structure and dynamics of proteins relate to their function has challenged scientists for several decades but still remains open. Conformational exploration methods aim to characterize the conformational space of proteins in order to find minimum energy regions corresponding to highly populated structures [1, 2]. These intermediate states are transient and therefore hard to detect experimentally. However, they may be crucial to understanding dynamic events such as folding, docking, binding and conformational change processes. The potential energy landscape of a protein is often rugged and has a large number of local minima [3]. This makes it difficult to navigate. The problem becomes even more challenging due to the fact that a typical protein can contain several hundreds of amino acids or several thousands of atoms. Therefore, the search space made out of all possible conformations that a protein can assume is large and its enumeration is practically impossible. Existing physics-based computational methods that sample the conformational space of proteins include Molecular Dynamics (MD) [4], Monte Carlo (MC) [5] and their variants, as well as approximate methods based on geometric sampling [2, 6–8], Elastic Network Modeling [9, 10], normal mode analysis [11, 12], morphing [13] and several other methods.

Even after the conformational space is sampled, it should be filtered and clustered to extract meaningful information. Several clustering methods have been designed for protein conformational space [6, 14, 15]. The majority of clustering methods for high-dimensional data incorporates metric functions that evaluate the distance between objects in the dataset, or a lower-dimensional representation of these objects, often trying to detect outliers [16].

Hierarchical clustering methods result in a multi-scale view of the conformational space and enable us to view the hierarchical relationship between the local minima produced by the conformational search. In this work we use both algebraic topology and dimensionality reduction methods to explore and characterize the conformational space of proteins. Algebraic topology has been used in the past for clustering data [17] and exploring the conformational space of small peptides, including finding metastable states [15]. In previous work [18, 19] we used persistent homology to explore the conformational space of proteins and detect regions of interest that may correspond to local minima, which are hard to detect experimentally due to the relatively short time the protein spends in them. We used only standard Principal Component Analysis (PCA), whose linear nature may not capture the complex, non-linear nature of the conformational spaces. Standard PCA is also known to be highly sensitive to outliers. Additionally, we selected the clustering parameters based on *empirical* observation. In contrast, in this paper we tested several dimensionality reduction methods to see which ones yielded the best projection for clustering. From all the methods we tested, sphPCA and Isomap (both non-linear) gave us better results. Other dimensionality reduction methods may also be suited for clustering, and we plan to revisit this in future work. In this paper we employ hierarchical clustering to detect intermediate states in the conformational space. The main contributions of this work are as follows:

1. Our parameter choice is automated and based on the properties of the input data, reducing the dependency on user-defined parameters.
2. The hierarchical clustering allows us to view the data from multiple resolutions, detecting intermediate states at a coarser or finer level, at our choice.
3. The clustering is done in the reduced space, thus avoiding the high computational cost of clustering high-dimensional data. Despite that, our clusters are very well-defined even when measuring their properties in the full structural space. (see Tables 1 and 2.)

**Table 1:** Isomap cluster analysis for Calmodulin, AdK and GroEL. The data is visualized in Fig. 3

Cluster No.	Size	RMSD (1CTR)	RMSD (1CLL)
1	10	14.71±0.2	1.95±0.2
2	5	13.89±0.1	2.69±0.1
3	22	13.43±0.5	3.82±0.7
4	19	10.23±1.0	6.88±0.9
5	5	7.97±0.3	8.43±0.3
6	47	3.86±1.9	11.99±1.7
Cluster No.	Size	RMSD (1AKE)	RMSD (4AKE)
1	15	6.49±0.2	1.99±0.2
2	19	6.04±0.2	2.85±0.3
3	6	4.96±0.3	3.73±0.1
4	8	4.65±0.5	3.84±0.2
5	10	3.56±0.2	4.42±0.2
6	33	2.91±0.6	5.91±0.6
Cluster No.	Size	RMSD (1SX4)	RMSD (1SS8)
1	11	11.56±0.2	2.50±0.6
2	13	11.22±0.2	3.95±0.5
3	20	10.40±0.5	5.25±0.5
4	8	8.34±0.5	6.58±0.4
5	29	5.46±1.2	8.85±0.7
6	17	2.39±0.9	11.35±0.6

The RMSD is measured by the cluster geometric center with respect to each one of the end points. The clusters numbers are sorted according to their RMSD (in Å) with respect to their original endpoints

**Table 2:** Spherical-PCA cluster analysis for Calmodulin, AdK and GroEL

Cluster No.	Size	RMSD (1CLL)	RMSD (1CTR)
1	14	14.63±0.3	2.04±0.4
2	8	13.28±0.4	4.40±0.4
3	6	11.89±0.5	5.60±0.3
4	13	9.55±1.0	7.45±0.6
5	41	2.73±1.4	12.96±1.2
Cluster No.	Size	RMSD (1AKE)	RMSD (4AKE)
1	26	6.28±0.3	2.41±0.5
2	47	2.56±1.4	5.55±1.2
3	5	2.36±0.2	6.00±0.1
Cluster No.	Size	RMSD (1SX4)	RMSD (1SS8)
1	25	11.42±0.3	3.17±0.8
2	14	10.20±0.4	5.31±0.5
3	39	3.74±1.4	10.24±1.2

The RMSD is measured similar to Table 1

At first sight, our results seem to be limited by the number of data points or the choice of landmarks. However, persistent Homology is robust enough so that the results do not depend on these, as explained in [20, 21].

Finally, as we pointed out earlier, Isomap generally performs better than PCA, at least on the examples presented. One reason is because, a priori, the topology of the original space will be different to that of the PCA-reduced space, since this is given by projection, and in general projections will distort the topology. In contrast, Isomap gives an embedding, which preserves topological features on the components on which the embedding is defined.

## Methods

### Conformational search

Table 3 shows the proteins used in this work. Each conformational pathway was modeled using a Monte-Carlo (MC) based search described below. Due to the size of the proteins, a fully atomic representation of the structure is computationally costly. Therefore, the proteins were represented using their C- *α* atoms, and their potential energy was estimated using a C- *α* based energy function [22]. The search was run for a maximum of 60,000 iterations. This number was determined experimentally. At every iteration a parent protein conformation is chosen from the conformation pool, then a rotatable bond between two C- *α* atoms is selected with a probability linearly proportional to the difference between this angle and its counterpart in the goal conformation, which serves as a bias of the search. Similar angles between start and goal conformation are skipped. The selected angle was rotated by a random value between -5 and 5 degrees. The new conformation is considered further only if its energy is below a threshold. The RMSD of the new conformation with respect to the goal, RMSD_new_, is calculated and compared to that of parent conformation, RMSD_parent_. The new conformation is accepted and added to the conformation pool according to the Metropolis criterion, if either of the following occurs:

**Table 3:** Proteins used in this study. The PDB ids of two known structures of each protein are listed

Protein	Calmodulin I	AdK	GroEL
No. Amino acids	144	214	524
Structure 1	1CLL	1AKE	1SS8
Structure 2	1CTR	4AKE	1SX4
RMSD	14.84	7.13	12.21
No. Clusters (Isomap)	6	6	6
No. Clusters (PCA)	5	3	3

1. |RMSD_new_|<|RMSD_parent_|
2. $\ln r < -\frac {|\text {RMSD}_{\operatorname {new}}|-|\text {RMSD}_{\operatorname {parent}}|}{a|\text {RMSD}_{\operatorname {new}}|}$,

for a scaling factor *a*, and *r* the probability of the new conformation. The final result is a pathway leading from the start conformation to the goal conformation. We generated 9543 sampling data points for Calmodulin (1CLL →1CTR), 7519 data points for AdK (1AKE →4AKE) and 11,038 data points for GroEL (1SS8 →1SX4).

### Data representation and PCA methods

The data was represented using several dimensionality reduction methods, which we now describe for completeness.

***Robust PCA methods:*** Standard PCA methods are sensitive to the presence of outliers in the data set. Attempts to overcome this sensitivity are robust generalizations of these linear PCA methods, specifically implemented to either remove outliers or diminish the errors produced by outliers. A non-linear robust dimensionality reduction method is *spherical* PCA (sphPCA), where the data is scaled so that each data vector is unitary and then one applies standard PCA to the new rescaled data to obtain the principal directions. This method reduces the influence of outliers as explained in [23]. In this paper we use sphPCA, which we found was the most efficient for hierarchical clustering.

***Isomap:*** Isomap is a non-linear dimensionality reduction method that uses multi-dimensional scaling (MDS) [24]. The Isomap algorithm estimates the distances of neighbors using geodesics via a weighted graph, constructed using a *K*-nearest neighbor search to connect the data points, thus preserving linearity only in the small neighborhood of each point (tangent space). In this work we used the minimal K to generate one connected component. Then, a standard algorithm to obtain the shortest path (geodesic) between two vertices in the graph is performed. Finally, this matrix is subject to MDS, extracting reaction coordinates which determine the embedding.

### Conformation space homology, algorithms and generators

Quantitative analysis of the conformational space can be done using Algebraic Topology methods. This enables us to study the global properties of conformational spaces as well as to detect rigid local properties, as is the number of local minima in a protein conformational space, by using a natural stratification of the space by energy level. This is, for a given energy *e*, we consider the subsets of conformations *X* ^≤*e*^ with energy bounded by *e*, then $X^{\le e_{1}}\subset X^{\le e_{2}}, $ for *e*_1_≤*e*_2_. The topology of such subsets will change as the parameter *e* increases and thus, local minima can be detected. In general, even when the space is given in closed form (by equations), its topology is difficult to determine. In our approach, the spaces are sets of data points generated by the sampling algorithm and thus we have computational restrictions as well. However, we will see that the lower dimensional homology can be determined experimentally.

**Fig. 1:**
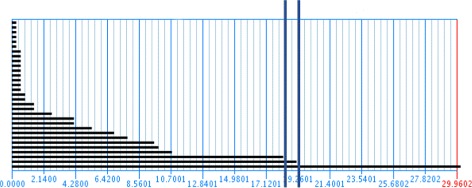
An example of a barcode diagram. The point where three clusters merge into two, and two merge into one are marked by vertical bars

Let us describe the topological tools used in our previous work [18]. Suppose *X* is the (continuous) set of low-energy conformations of a protein, projected to some lower-dimensional metric space. We equip *X* with the natural *topology* it inherits from the ambient space. We let *H*_*k*_(*X*) denote the simplicial homology (with coefficients in a fixed field [25]). We will denote by *b*_*k*_ the *k*-th Betti number of *X*, i.e. the dimension of *H*_*k*_(*X*) as a vector space. The 0-th Betti number *counts* the number of connected components or pieces of the space, since any two points are homologous if and only if they are a boundary of a 1-chain. In this paper *X* will be in general approximated by a discrete set of sample points *Z*⊂*X*, which is obtained from the conformational search algorithm. We extract information of *X* from *Z*, using Persistent Homology [26] to estimate the topology of *X* through algorithms applied to the approximation *Z*. Persistent homology has been successful in detecting topological features of data sets, see for instance [27]. Computational algorithms to obtain persistent homology are described in [20]. In this paper we not only estimate the Betti numbers of *X*, we are interested in finding geometric generators for the homology from the original set. The tools used in the paper are well-suited for data sets. Given a real number *r*>0, we let *C*_∗_(*Z,r*) be the simplicial complex whose set of vertices is the set *Z* itself. We declare the *k*-simplexes as the sets {*x*_0_,…,*x*_*k*_}⊂*Z* if the distance *d*(*x*_*i*_,*x*_*j*_)≤*r* for all *i*≠*j*. The boundary is composed by the maps forgetting one of the vertices. The value of *r* that we should use to detect the actual topological information of the space *Z* is initially unknown. The parameter *r* defines a *stream* of complexes: for each *r*_1_<*r*_2_ we have *C*_∗_(*Z,r*_1_)↪*C*_∗_(*Z,r*_2_), and thus we get natural maps *H*_*k*_(*C*_∗_(*Z,r*_1_))→*H*_*k*_(*C*_∗_(*Z,r*_2_)) for all *k*. This yields a sequence of vector spaces *H*_*k*_(*C*_∗_(*Z,r*)), and their dimensions *b*_*k*_(*r*) yield *bar codes* associated to *Z*, one for each *k*, which encode the evolution of generators of each cohomology (and thus *b*_*k*_) as *r* increases (See Fig. 1). These barcodes are formally a set of intervals of the real line, bounded below. A long line in the *k*-th barcode means that there is a *k*-cycle that persists as *r* increases, and thus it detects an *actual* generator of the homology of the original space *X*. All small bars are not persistent and are considered noise. We will assume that *r* will eventually be large enough, say *r*_max_, so that *Z* is covered by balls of radius *r*_max_, and thus the complex collapses. We are interested in tracing back the generator for each long bar.

### Lazy witness streams and landmarks

The actual algorithm for computing the bar-codes is a modification of the description above, using landmark points and *lazy witness streams.* [21]. We specify a set *Z*_0_ of *landmarks* in *Z*. *Z*_0_ is selected according to a sequential mini-max scheme. The first landmark is picked randomly in *Z*. Inductively, if *Z*_0_(*i*−1) is the set of the first *i*−1 landmarks, then we let the *i*-th landmark point to be the point of *Z* which maximizes the Euclidean distance *d*(*z,Z*_0_(*i*−1)) between the point *z* and the set *Z*_0_(*i*−1). This scheme provides better coverage of the point cloud than a random selection of the landmark points [28]. Given *z*∈*Z*, we consider the set of all distances {*d*(*z,y*),*y*∈*Z*_0_} from *z* to *y*∈*Z*_0_, and we order them. We denote by *d*_*k*_(*z*) the *k*-th term, that is the distance from *z* to its (*k*+1)-closest landmark point. The *witness stream complex* *W*_*k*_(*Z,Z*_0_,*t*) has vertices (*k*=0) *Z*_0_ and for *k*>0, a *k*-simplex {*z*_0_,…,*z*_*k*_} is in *W*_*k*_(*Z,Z*_0_,*t*) if all of its faces (subsets of cardinality *k*) are in *W*_*k*−1_(*Z,Z*_0_,*t*) and if there is a *witness* *z*∈*Z* (which could be in *Z*_0_) for which max{*d*(*y,z*_*i*_),*i*=0,…,*k*}≤*t*+*d*_*k*_(*z*). This clearly defines a stream depending on *t*, since for *t*_1_<*t*_2_, *W*_∗_(*Z,Z*_0_,*t*_1_)→*W*_∗_(*Z,Z*_0_,*t*_2_) is an inclusion. Computationally, a witness stream is still quite expensive and thus other modifications are used to estimate Betti numbers. For an integer parameter *ν*≥0, the *lazy* witness complex $LW^{\nu }_{*}(Z,Z_{0},t)$ is the stream defined as follows. For *z*∈*Z*, let *d*(*z*) denote the distance from *z* to the *ν*-closest point in *Z*_0_, just as we did before. Now, define $LW^{\nu }_{0}(Z,Z_{0},t)$ as the set *Z*_0_. An edge {*z*_0_,*z*_1_} is in $LW^{\nu }_{1}(Z,Z_{0},t)$ only if there is a witness *y*∈*Z* such that max{*d*(*y,z*_0_),*d*(*y,z*_1_)}≤*t*+*d*(*y*). The *k*>1 simplex {*z*_0_,…,*z*_*k*_} is in $LW^{\nu }_{k}(Z,Z_{0},t)$ if all of its edges are. The lazy witness complex $LW^{\nu }_{*}(Z,Z_{0},t)$ is a *flag* complex, that is, it is entirely determined by its 1-dimensional skeleton (graph). Note that this modification does not affect the estimation of connected components, but it does depend on *ν*. We use either *ν*=0,1, however to detect an actual generator of of 0-th homology (long bar) we use *ν*=0. Once a long bar is identified, we find all of the points corresponding to the component using a union-find algorithm, compatible with the Javaplex internal data structures. We then evaluate the cluster that corresponds to these points.

### Hierarchical clustering

To elucidate the topology of the conformational space and to detect intermediate structures we estimated the location of highly populated clusters based on the intervals obtained by the barcodes. The input is the lower-dimensional of the coordinates of a conformational trajectory. We first set out to determine the appropriate number of landmarks for every sample. The choice of landmarks is important to provide sufficient coverage of the conformational space on one hand, and to avoid over-fitting on the other hand. For each sample we ran Javaplex successively with 10,20,30,*e* *t* *c*. landmarks, and measured the variation in *R*, the maximum distance of a point from a landmark. We stop when the difference in *R* between two consecutive runs is less than 5%. This means that adding more landmarks does not affect the coverage significantly. To account for the randomness in the selection of landmarks, we averaged the resulting number of landmarks over 5 runs and used the average + 2 standard deviations. For all the examples in this paper approximately 100 landmarks proved sufficient. Some of these landmarks were outliers and were removed during the clustering. Note that for the method used in this paper, we always use the same set of landmarks in all the JavaPlex runs of the same data set, for consistency.

We then determined the number of clusters systematically by running Javaplex, using the number of landmarks as determined above. The “natural” number of clusters is hard to determine in the general case, but the following heuristic turned out to work well. We successively ran javaplex on the set of coordinates and set the radius *r* to generate *i* bars in each run, where *i*={1,2,…20}. The number of clusters was increased by 1 whenever the difference between two consecutive bars in the barcode plot was more than 0.1 in the barcode plot when *b*_0_ is calculated. We used the same set of landmarks at each run to make sure the topology of the conformational space and the computational setup is the same each time. To determine the hierarchy between the clusters we checked which cluster split by testing which two sets of landmarks constituting two clusters in the (*i*+1)^*st*^ run were a proper subset of a cluster in the *i* ^*th*^ run. This hierarchy between clusters can be displayed using a dendrogram which traces back the relationship between clusters generated by consecutive runs. The clusters are then traced back to the original conformations from the full coordinate space.

## Results and discussion

For each selected protein, the C- *α* based representation of the start and target points are used to obtain the conformations pathways that link the two end points by using the MC-based method described in the “Methods” section above. The number of amino acids of each protein is 144 for Calmodulin, 214 for AdK and 524 for GroEL (See Table 3). The dimensionality of the conformational space, representing each conformation by a 3×*N* vector with the *x,y,z* coordinates of each C- *α*, is therefore 432 for Calmodulin, 642 for AdK and 1572 for GroEL. However, the “true” underlying dimensionality for protein structures is much smaller than the number of atomic coordinates requires to represent their structures, due to mutual constraints and interactions between different parts of the proteins. One can see this by computing the variance of the data in the reduced representation. When running sphPCA on the conformational spaces of all the proteins, the first three dimensions account for 90% or more of the variance in the data. For Isomap, three dimensions explain >99% of the variance in the data. The first three coordinates are therefore used in all cases for comparison purposes.

### Cluster analysis

The cluster numbers below are assigned in increasing order of RMSD with respect to one of the endpoints. Table 3 shows the number of clusters detected for each test case. Below, we detail the results for each one of the tested systems.

**Fig. 2:**
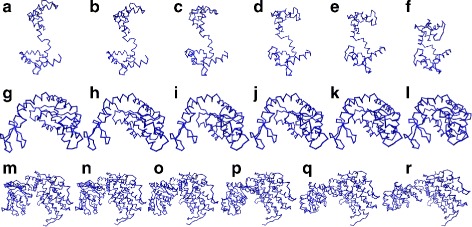
Representatives of the six cluster centers generated by Isomap for the first case of (**a**-**f**) Calmodulin (1CLL →1CTR). The centers are sorted according to their RMSD from 1CTR. (**g**-**l**) AdK. The clusters are sorted according to their RMSD from 1AKE. (**m**-**r**) GroEL. The clusters are sorted according to their RMSD from 1SX4

**Calmodulin:** The Isomap embedding produced six clusters based on the selected landmarks. One hundred eight landmarks were retained, and the rest discarded as outliers. Table 1 shows each cluster’s RMSD with respect to each of the two endpoints, 1CLL and 1CTR. As seen, the clusters span the conformational space between the two end points and the clusters are compact (with small standard deviation of RMSD around the geometric average). The clusters are numbered from 1 to 6 in reverse order of their RMSD with respect to 1CLL (see Table 1). Even though the clusters were numbered according to their RMSD from 1CLL, they are also sorted perfectly with respect to their RMSD from 1CTR. This shows that the clusters span the conformational space between the two endpoints. The distribution of the RMSDs of the cluster centers is not completely uniform, which is probably due to the sampling method which biases the search towards the goal structure. Obtaining more uniform sampling is the subject of current work. Figure 2 a-f shows the six cluster representative structures, sorted by their RMSD from 1CLL.

**Fig. 3:**
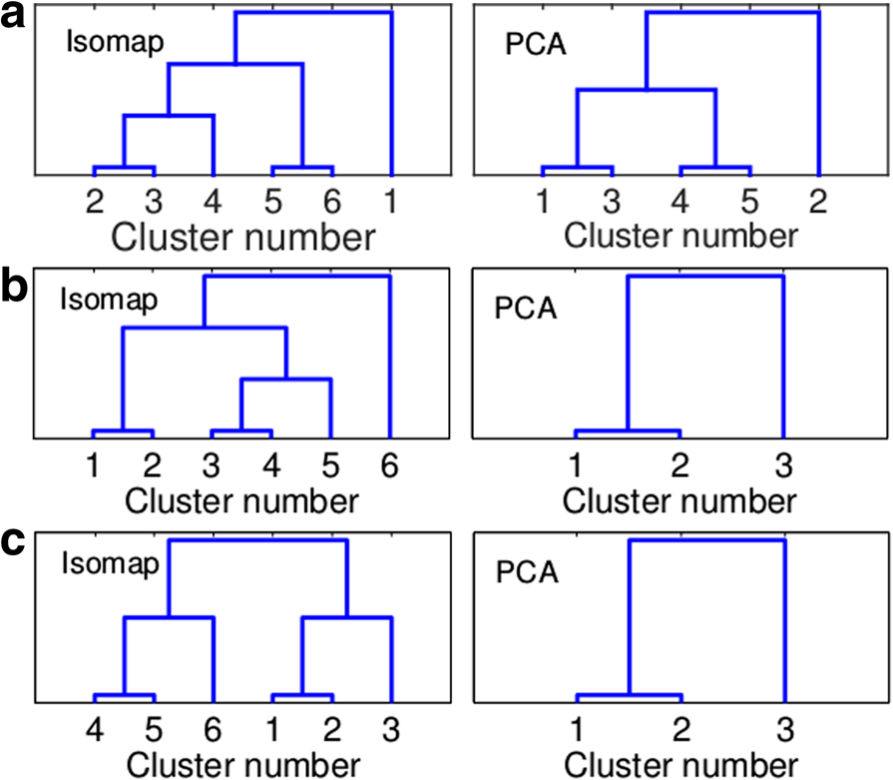
The hierarchical clustering structure for (**a**) Calmodulin (1CLL →1CTR) (**b**) AdK (1AKE →4AKE) (**c**) GroEL (1SS8 →1SX4). The plot shows the Isomap generated hierarchy (left) and the Spherical PCA hierarchy (right). The clusters are numbered by their RMSD from the end point

**Fig. 4:**
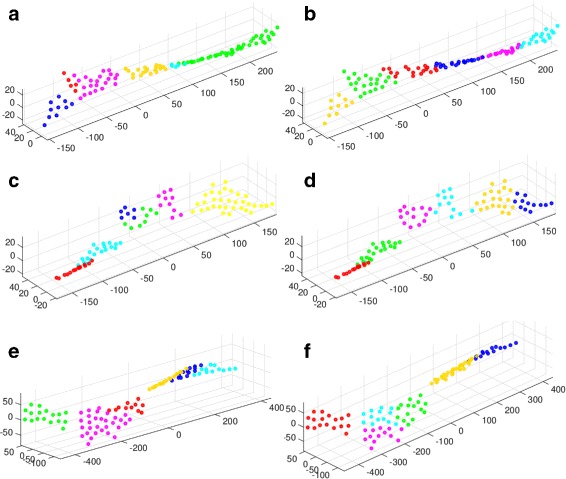
The projection of the clusters for (**a**-**b**) Calmodulin (1CLL →1CTR) (**c**-**d**) AdK (1AKE →4AKE) (**e**-**f**) GroEL (1SS8 →1SX4) The left plot shows the hierarchical clustering and the right plot shows the clusters generated by k-means. In each case the Isomap projection along the first three reaction coordinates is used. Every cluster is depicted in a different color

Figure 3 a illustrates the hierarchy of the clusters for both Isomap and Spherical PCA. Note that the height of the bars is arbitrary (the hierarchy is not, of course). For example – clusters 2 and 3 split from each other at the lowest level, and so are clusters 5 and 6. However, clusters 2 and 3 are much more similar to one another than clusters 5 and 6, as can be seen both visually in Fig. 2 and from their RMSD to the two end points in Table 1. The average RMSD of the entire set of landmarks is 9.75Å from 1CLL and 6.15Å from 1CTR. As seen in Table 3, the RMSD of the two endpoints is 14.84Å. Figure 4 a shows the Isomap projected landmarks where each resulting cluster is depicted in a different color

The sphPCA analysis resulted in five clusters. The hierarchical relationship and RMSD analysis can be shown in Fig. 3 a and on Table 2. As before, the cluster numbers were assigned with respect to the RMSD from 1CLL. The PCA clusters are generally less compact and cover less of the conformational space than in the Isomap case. It can be seen especially when examining Table 2. The distribution of the clusters is less uniform in the case of PCA, missing parts of the conformational space.

**Adenylate Kinase (AdK):** Six clusters were detected for AdK, using Isomap. Ninety-one landmarks were retained. The hierarchical relationship and the RMSD analysis of the clusters are shown in Table 1 and Fig. 3 b. The cluster numbering was assigned according to the RMSD from 1AKE. Figure 2 g-l shows the six cluster representative structures, sorted by their RMSD from 1AKE. Figures 4 c shows the Isomap projected landmarks where each cluster is depicted in a different color. As before Table 1 show that the clusters span the entire conformational space even when the RMSD is measured with respect to the other endpoint, 4AKE. The spherical PCA analysis resulted in three clusters. The hierarchical relationship and RMSD analysis can be shown in Fig. 3 b and on Table 2. As before, the cluster numbers were assigned with respect to the RMSD from 1AKE. As is the case with Calmodulin, The PCA clusters are generally less compact and cover less of the conformational space than in the Isomap case. Only three clusters were detected this time. For AdK there are known intermediate structures, and further validation is shown below.

**GroEL:** The Isomap analysis for GroEL produced six clusters containing 98 of the landmarks. The hierarchical relationship and the RMSD analysis of the clusters are shown in Fig. 3 c as well as in Table 1. The cluster numbering was assigned according to the RMSD with respect to 1SX4 in descending order. As before, It can be seen in Table 1, that the clusters span the entire conformational space even with respect to the other endpoint, 1SS8. Figure 2 m-r shows the six cluster representative structures, sorted by their RMSD from 1SX4. Figure 4 e shows the Isomap-projected landmarks where each cluster is depicted in a different color. The generated clusters are less compact than the clusters generated above for Calmodulin and AdK. However, this can be expected since GroEL is a big protein and its conformational transition seems to be more complex than the other two examples.

### Validation against known intermediates

Experimental validation is often difficult to obtain due to lack of experimental knowledge about intermediate structures. However, AdK has several known mutants and intermediate structures [29]. We focused on the following known intermediates: chains A, B, and C of the hetero-trimer Adenylate Kinase from Aquifex Aeolicus (PDB code 2RH5), which are conformational change intermediates of the ligand free AdK [30], 1E4Y, which is an AdK mutant having 99% sequence identity with 4AKE and 1AKE and is a closed form of AdK binding with AP5A, a form of AdK from Bos Taurus (PDB code 1AK2), and a mutant ligated with an ATP analog (PDB code 1DVR). These intermediates have been used successfully to validate conformational pathways for AdK [2, 8, 14, 31]. For each path, we recorded the closest conformation to any of our intermediates. The results are shown in Table 4. For each intermediate, the table shows the average RMSD from the closest cluster. Our results for Isomap are in good agreement with previous work [29], as well as our earlier studies [14, 32], which predicted 2RH5A-C to be close to the open conformation and 1E4Y to be closest to the closed conformation. Other structures are closer to intermediate conformations. Both Isomap and Spherical PCA were able to find intermediate structures close to 5 intermediates (within about 3Å or less). However, since Spherical PCA only produced three clusters, it is hard to tell whether the cluster centers span much of the conformational space.

**Table 4:** Comparison of clusters of AdK to known intermediates

PDB	Isomap closest clust. (RMSD)	PCA closest clust. (RMSD)
1E4Y	Clust 1 (1.7Å)	Clust 1 (2.1Å)
1AK2	Clust 2 (3.5Å)	Clust 1 (4.1Å)
1DVR	Clust 2 (2.6Å)	Clust 1 (2.8Å)
2RH5A	Clust 6 (2.2Å)	Clust 3 (2.5Å)
2RH5B	Clust 6 (2.3Å)	Clust 3 (2.4Å)
2RH5C	Clust 6 (3.0Å)	Clust 3 (3.1Å)

For every known intermediate, the RMSD to the closest cluster is shown. The cluster numbers are as in Fig. 2

### Comparison to K-means clustering

In order to validate our clustering algorithm, we compared our results to others generated by the K-Means algorithm [33]. K-means is a standard and well-known clustering method, and it is simple to implement. We used the Matlab K-means implementation. For the sake of comparison with our method, we generated six clusters for each one of the Isomap embeddings, which is the same number of clusters produced for each of our examples. As expected, K-means tends to produce roughly equidistant, similar sized clusters, so the results tend to be different than our connected components, which may vary significantly in size and distribution around the center. The K-means clustering of the Isomap data for CaM, AdK and GroEL is shown in Fig. 4 b, d, f, respectively. The hierarchical clustering for the Isomap data for CaM, AdK and GroEL is shown in Fig. 4 a, c, e, respectively. We placed the two clustering methods next to one another for visual comparison. It is difficult to estimate which structures represent “true” intermediates, especially due to the scarcity of experimental information and the coarse-grained nature of this search. However, the main advantage of our method is that unlike K-means we can easily detect outliers and we can more easily determine the number of clusters. Additionally, the similar sizes and symmetric cluster shapes produced by K-means may produce a bias against the topology and shape of the conformational space.

## Conclusions

Many proteins undergo large-scale conformational changes as part of their function. Characterizing the conformational space of proteins is crucial for understanding their function and dynamics. Finding intermediate conformations which may correspond to local minima is important but highly challenging due to these conformations being transient and the lack of experimental data about intermediate states. We present a persistent homology and dimensionality reduction based hierarchical method to detect clusters of intermediate structures in the conformational spaces of proteins undergoing large-scale changes. The method is able to produce compact clusters that span the conformational spaces of the sampled proteins, and the hierarchical clustering allows us to obtain a multi-scale view. We use a projection to a low-dimensional subspace that preserved the variance in the data. This projection is the input to the persistent-homology based hierarchical clustering, from which intermediate structures are extracted. We tested two non-linear dimensionality reduction methods – Isomap and sphPCA. We find that in general Isomap provides more compact and robust clusters. Future work includes energetic filtering that will allow us to detect high-energy barriers and low-energy folding pathways and domain motions. We also plan to obtain a comprehensive characterization the conformational landscapes of smaller peptides using trajectories produced by all-atom MD simulations.
